# Toward Personalized Withdrawal of TNF-α Inhibitors in Non-Systemic Juvenile Idiopathic Arthritis: Predictors of Biologic-Free Remission and Flare

**DOI:** 10.3390/ph19010125

**Published:** 2026-01-10

**Authors:** Ekaterina I. Alexeeva, Irina T. Tsulukiya, Tatyana M. Dvoryakovskaya, Ivan A. Kriulin, Dmitry A. Kudlay, Anna N. Fetisova, Maria S. Botova, Tatyana Y. Kriulina, Elizaveta A. Krekhova, Natalya M. Kondratyeva, Meiri Sh. Shingarova, Maria Y. Kokina, Alyona N. Shilova, Mikhail M. Kostik

**Affiliations:** 1 Department of Pediatric Rheumatology, National Medical Research Center of Children’s Health, 119991 Moscow, Russia; alekatya@yandex.ru (E.I.A.); irinatsulukiya@gmail.com (I.T.T.); tbzarova@mail.ru (T.M.D.); 79671819676@yandex.ru (I.A.K.); anna_534@mail.ru (A.N.F.); mariabotova22@gmail.com (M.S.B.); t.kriulina@aspirre-russia.ru (T.Y.K.); lizakrek@mail.ru (E.A.K.); 131nk@mail.ru (N.M.K.); mshingarova@mail.ru (M.S.S.); kokinamariah@yandex.ru (M.Y.K.); alin23flu23@gmail.com (A.N.S.); 2 Clinical Institute of Children’s Health Named After N.F. Filatov, Department of Pediatrics and Pediatric Rheumatology, I.M. Sechenov First Moscow State Medical University (Sechenov University), 119435 Moscow, Russia; 3 Association of Pediatric Rheumatologists, 107078 Moscow, Russia; 4 Department of Pharmacology, Institute of Pharmacy, I.M. Sechenov First Moscow State Medical University (Sechenov University), 119435 Moscow, Russia; d624254@gmail.com; 5 Laboratory of Personalized Medicine and Molecular Immunology, National Research Center—Institute of Immunology of Federal Medical-Biological Agency of Russia, 115522 Moscow, Russia; 6 Hospital Pediatry, Saint-Petersburg State Pediatric Medical University, 194100 Saint-Petersburg, Russia

**Keywords:** juvenile idiopathic arthritis, tumor necrosis factorα inhibitors, biological disease-modifying antirheumatic drugs, biological treatment withdrawal, sustained remission, predictors, pediatric rheumatology

## Abstract

**Background:** Tumor necrosis factor-α (TNFα) inhibitors have significantly improved outcomes in children with non-systemic juvenile idiopathic arthritis (JIA), achieving long-term clinical remission for many patients. However, the optimal strategy for TNF-α inhibitor withdrawal remains unknown, whether through abrupt discontinuation, gradual dose reduction, or interval extension. **Objective:** We aim to identify patient-, disease-, and treatment-related predictors of successful TNF-α inhibitor withdrawal in children with non-systemic JIA. **Methods:** In this prospective, randomized, open-label, single-center study, 76 children with non-systemic JIA in stable remission for ≥24 months on etanercept or adalimumab were enrolled. At the time of TNF-α inhibitor discontinuation, all patients underwent a comprehensive evaluation, including a clinical examination, laboratory tests (serum calprotectin [S100 proteins] and high-sensitivity C-reactive protein [hsCRP]), and advanced joint imaging (musculoskeletal ultrasound and magnetic resonance imaging [MRI]) to assess subclinical disease activity. Patients were randomized (1:1:1, sealed-envelope allocation) to one of three predefined tapering strategies: (I) abrupt discontinuation; (II) extension of dosing intervals (etanercept 0.8 mg/kg every 2 weeks; adalimumab 24 mg/m^2^ every 4 weeks); or (III) gradual dose reduction (etanercept 0.4 mg/kg weekly; adalimumab 12 mg/m^2^ every 2 weeks). Follow-up visits were scheduled at 3, 6, 9, 12, and 18 months to monitor for disease relapse. **Results:** Higher baseline Childhood Health Assessment Questionnaire (CHAQ) scores (≥2), elevated serum calprotectin [S100 proteins] and hsCRP levels at withdrawal, imaging evidence of subclinical synovitis, and a history of uveitis were all significantly associated with increased risk of flare. No significant associations were found for other clinical or demographic characteristics. **Conclusions:** Early significant clinical response, absence of subclinical disease activity, and concomitant low-dose methotrexate therapy were key predictors of sustained drug-free remission. These findings may inform personalized strategies for biologic tapering in pediatric JIA.

## 1. Introduction

Juvenile idiopathic arthritis (JIA) is the most common chronic inflammatory rheumatic disease in children and a leading cause of acquired disability [1]. The introduction of biologic disease-modifying antirheumatic drugs (bDMARDs), particularly tumor necrosis factor-α (TNF-α) inhibitors such as etanercept and adalimumab, has transformed disease management, substantially improving remission rates and long-term functional outcomes in non-systemic JIA [2].

With the widespread use of TNF-α inhibitors, an increasing proportion of patients now achieve sustained clinical remission. Nevertheless, the optimal strategy for treatment withdrawal remains uncertain—whether through abrupt discontinuation, gradual dose reduction, or interval extension. Premature withdrawal may trigger disease flare and irreversible structural damage, while unnecessarily prolonged therapy exposes patients to adverse events and considerable economic burden [3].

Recent studies highlight the importance of individualized approaches to biologic tapering in JIA. Incorporating clinical characteristics, laboratory biomarkers, and advanced imaging may enhance the prediction of relapse risk and facilitate more precise treatment decisions [3].

The present study aimed to identify clinical, laboratory, and imaging predictors of sustained biologic-free remission after TNF-α inhibitor discontinuation in children with non-systemic JIA. By addressing this knowledge gap, we aimed to provide evidence that can guide personalized therapeutic strategies and enhance long-term outcomes in pediatric patients.

## 2. Results

### 2.1. Demographic and Baseline Characteristics

A total of 76 children with non-systemic JIA were included in the analysis, comprising 47 females (61.8%) and 29 males (38.2%). The median age at disease onset was 3.6 years (interquartile range [IQR], 1.8–5.8 years).

The distribution of JIA categories was as follows: oligoarticular JIA was the most common subtype (42/76, 55.2%), including 26 patients (34.2%) with persistent and 16 (21.0%) with extended oligoarthritis. Polyarticular JIA was diagnosed in 17 patients (22.3%), and enthesitis-related arthritis (ERA) in another 17 (22.3%). A history of uveitis was reported in 8 patients (10.5%).

*HLA-B27* and ANA testing were performed in all patients. *HLA-B27* positivity was observed in 18 out of 76 patients (23.7%), while ANA was detected in 42 out of 76 patients (55.3%).

Notably, none of the patients with RF-positive polyarticular JIA achieved sustained remission for 24 months and were therefore excluded from the cohort.

At the time of TNF-α inhibitor withdrawal, 45 patients (59.2%) were receiving concomitant non-biologic DMARDs, predominantly methotrexate (43/45, 95.6%). Leflunomide and cyclosporine were used in one patient (2.2%). Following randomization, 23 patients (30.3%) discontinued DMARD therapy during follow-up.

The majority of patients (71/76, 93.4%) were biologic-naïve before starting TNF-α inhibitors. Four patients (5.6%) had previously received a non-TNF biologic but were switched to etanercept or adalimumab due to inadequate response. Etanercept was the most frequently used TNF-α inhibitor (67/76, 88.2%), while nine patients (11.8%) received adalimumab.

TNF-α inhibitor therapy was initiated at a median of 19 months (interquartile range [IQR]: 9–45) after disease onset. The median duration of TNF-α therapy was 54 months (IQR: 40–74), with a median duration of clinical remission on treatment of 46 months (IQR: 33–67) before withdrawal.

### 2.2. Assessment of Subclinical Disease Activity Prior to TNF-α Inhibitor Withdrawal

All patients underwent a comprehensive evaluation for subclinical disease activity prior to discontinuation of TNF-α inhibitors. Elevated markers of subclinical inflammation, defined as serum S100 protein levels greater than 2.9 µg/L and/or high-sensitivity C-reactive protein (hsCRP) levels greater than 5 mg/L, were detected in 17 of 76 patients (22.4%). The median serum S100 protein concentration was 1.18 µg/L (IQR: 0.90–1.81), and the median hsCRP level was 0.5 mg/L (IQR: 0.1–1.05). Subclinical synovitis of at least one previously affected joint was identified by the joint US or MRI in 45 of 76 patients (59.2%). Synovial hyperplasia was visualized on ultrasound in 5 of 76 patients (6.6%) and on MRI in 6 of 76 patients (7.9%). MRI additionally revealed tenosynovitis in 5 of 76 patients (6.6%) and bone marrow edema in 11 of 76 patients (14.5%). Demographic, disease characteristics, and treatment of the enrolled patients are in Table 1.

### 2.3. Flare Outcomes

**Figure 1:**
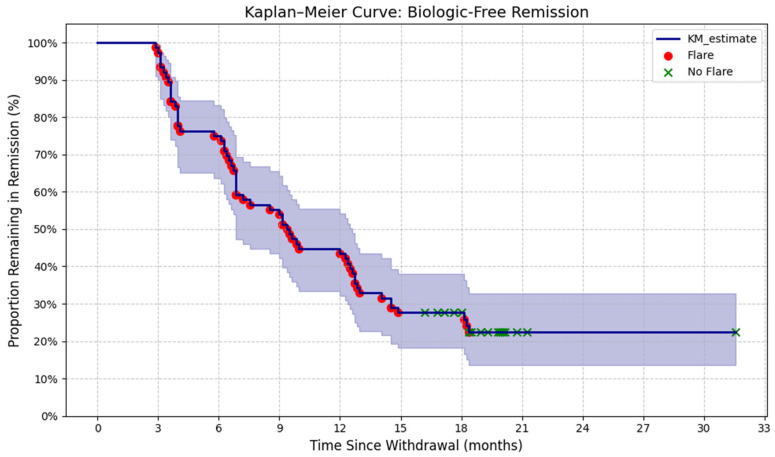
Kaplan–Meier analysis of biologic-free remission duration.

**Figure 2:**
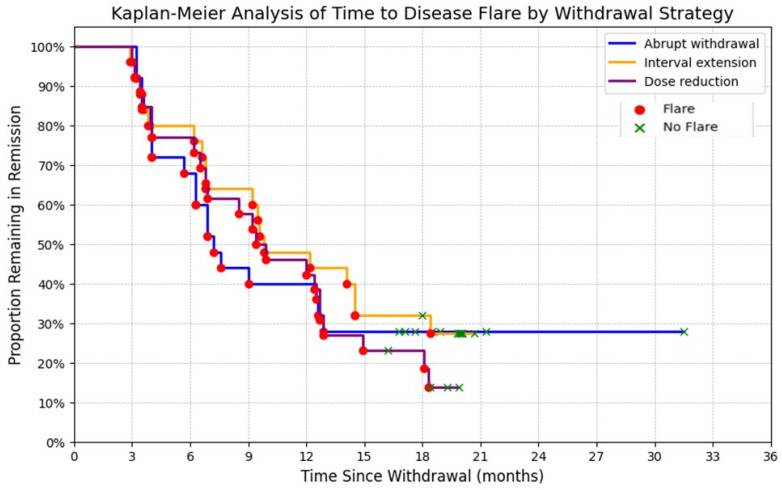
Kaplan–Meier survival curves for time to disease flare after discontinuation of different TNF-α inhibitors.

The majority of patients (58/76, 76.3%) experienced a disease flare after TNF-α inhibitor withdrawal. Flare rates by group were as follows: Group I (abrupt withdrawal): 18/25 patients (72.0%); Group II (gradual interval extension): 18/25 patients (72.0%); Group III (gradual dose reduction): 22/26 patients (84.6%). There were no statistically significant differences in flare rates among the three withdrawal strategies (*p* > 0.05). The median time to flare for the overall cohort was 12.4 months (IQR 7.7–12.9; range 2.0–31.5 months). Median duration of drug-free remission by group was: Group I: 12.6 months (IQR 7.0–18.0); Group II: 10.0 months (IQR 6.9–19.3); Group III: 12.6 months (IQR 6.9–15.1). No significant differences in time to flare were observed between groups. A total of 18 patients (23.6%) remained in sustained (≥12 months), drug-free remission without flare at a median follow-up of 19.7 months (IQR, 18.0–20.4; range, 16.2–31.5 months), as assessed by the Wallace criteria. Kaplan–Meier curve showing time to disease flare after TNF-α inhibitor withdrawal in non-systemic JIA. Most flares occurred within 12 months, with a small proportion maintaining long-term remission. (Figure 1 and Figure 2).

### 2.4. Clinical Characteristics of Flares Following TNF-α Inhibitor Discontinuation

Among the 58 patients who experienced a flare after TNF-α inhibitor withdrawal, the majority presented with arthritis alone (50/58; 86%), while uveitis flares occurred in 7 patients (12%) and a combined flare of arthritis and uveitis in 1 patient (2%). When analyzed by withdrawal strategy, in the abrupt withdrawal group (n = 18/25), flares manifested predominantly as arthritis (17 patients, 94%), with a single case of isolated uveitis (6%). In the interval extension group (n = 18/25), arthritis flares occurred in 13 patients (72%), uveitis in 4 patients (22%), and one patient (6%) developed a combined flare of arthritis and uveitis. In the dose reduction group (n = 22/26), arthritis was again the most common manifestation, occurring in 20 patients (91%), whereas uveitis was observed in 2 patients (9%).

The most commonly affected joints during arthritis flares were the knee (43/51; 84%) and the ankle (25/51; 49%). Less frequently involved joints included the hip in 2/51 (4%), the small joints of the hands and feet in 3/51 (6%), and the sacroiliac joints in 1/51 (2%). The median number of active joints at the time of flare was 2 (IQR 1–7), which was significantly lower than at disease onset (median 7, IQR 2–7; *p* < 0.001, Wilcoxon signed-rank test). Among patients with a history of uveitis (n = 8), flare occurred within 3 months in 1 patient (12.5%), 6 months in 4 patients (50%), 9 months in 1 patient (12.5%), and 12 months in 2 patients (25%). At the time of flare, systemic inflammation markers remained generally low. The median ESR was 11 mm/h (IQR 2–66), and the median CRP level was 5.7 mg/L (IQR 1–24). Elevated ESR (>20 mm/h) was observed in 4/58 patients (6.9%), and elevated CRP (>5 mg/L) in 7/58 patients (12%). For comparison, at disease onset, the median ESR was 18 mm/h (interquartile range [IQR], 6–28), and the median CRP was 6 mg/L (IQR, 2–15).

In 49 out of 58 patients with arthritis flare (84.4%), the pattern of joint involvement mirrored that seen prior to TNF-α inhibitor withdrawal. In 6 patients (10.3%), at least one newly affected joint was identified during flare.

### 2.5. Treatment Reintroduction After Disease Flare

Of the 76 patients who discontinued TNF-α inhibitor therapy following sustained disease control, 58 (76.3%) experienced a disease flare during follow-up. Among them, 56 patients (96.5%) were restarted on pharmacologic treatment by their treating pediatric rheumatology teams.

The majority of these patients (n = 54; 96.4%) resumed the same TNF-α inhibitor previously used (etanercept or adalimumab), while two patients (3.6%) were switched to an alternative biologic agent due to inefficacy. An additional two patients (3.4%) with mild disease reactivation were managed with non-biologic therapies alone (methotrexate or tofacitinib), without the need to restart biologic treatment.

All retreated patients subsequently achieved clinical inactive disease, as defined by the C. Wallace criteria.

### 2.6. Remission Predictors After TNF-α Inhibitor Withdrawal

These parameters were assessed longitudinally at key time points: at disease onset, at initiation of TNF-α inhibitor therapy, 6 and 12 months after initiation, and at the time of drug discontinuation.

Multivariate analysis identified the following as significant predictors of sustained drug-free remission:

- Female sex;
- Achievement of ≥90% improvement by ACRpedi criteria at both 6 and 12 months of TNF-α inhibitor therapy;
- Negative ANA at the time of TNF-α inhibitor initiation;
- Absence of *HLA-B27* antigen;
- Ongoing methotrexate therapy after biologic withdrawal.

In contrast, the following were significantly associated with disease flare after TNF-α inhibitor discontinuation:

- CHAQ score ≥ 2 (as reported by the parent) at disease onset;
- Elevated serum S100 protein and hsCRP at the time of withdrawal;
- Presence of subclinical synovitis on ultrasound or MRI in previously affected joints;
- History of JIA-associated uveitis.

Other evaluated parameters, including age, disease duration, baseline ESR/CRP, and joint distribution, were not statistically associated with long-term remission outcomes (Table 2).

For predictors of biologic-free remission, remission was coded as the dependent outcome (event = 1). For predictors of disease flare, disease flare was coded as the dependent outcome (event = 1). OR greater than 1 indicates an increased likelihood of the corresponding outcome.

## 3. Discussion

### 3.1. Flare Rates and Timing After TNF-α Inhibitor Withdrawal

Throughout this manuscript, sustained biologic-free remission refers to remission maintained for at least 12 months after TNF-α inhibitor withdrawal; earlier time points represent interim follow-up assessments.

In this prospective, randomized, open-label, single-center study of 76 children with non-systemic juvenile idiopathic arthritis (JIA) who had maintained clinical remission on TNF-α inhibitors for ≥24 months, disease relapse after treatment discontinuation was standard: 58 patients (76.3%) experienced a flare during follow-up, whereas 18 patients (23.6%) remained in sustained biologic-free remission at a median follow-up of 19.7 months (IQR 18.0–20.4). The median time to flare for the overall cohort was 12.4 months (IQR 7.7–12.9; range 2.0–31.5), and most relapses occurred within the first year after withdrawal. Flare rates by withdrawal strategy were 72.0% (18/25) after abrupt discontinuation, 72.0% (18/25) after interval extension, and 84.6% (22/26) after dose reduction; these differences did not reach statistical significance. These results corroborate the general pattern reported in the literature: biologic withdrawal in JIA often leads to relapse in the majority of patients, and the highest incidence is observed within the first 6–12 months [4,5]. The magnitude of relapse observed here (75%) aligns with prior studies that have documented high flare rates following cessation of anti-TNF therapy. However, exact estimates vary depending on the population, disease subtype, and tapering protocol [5,6]. Notably, the absence of statistically significant differences among withdrawal modalities suggests that the choice of tapering technique (abrupt vs. interval vs dose reduction) may be less determinative of outcomes than patient-specific characteristics and residual subclinical disease activity.

Interestingly, the median time to flare in our prospective study population (12.4 months) was shorter than that reported in some retrospective studies, in which sustained biologic-free remission lasted approximately 22 months [7]. This discrepancy may reflect differences in study design, patient selection, and the intensity of follow-up. Prospective monitoring enables more frequent and standardized assessments, which may capture flares earlier than retrospective chart reviews. Additionally, stringent inclusion criteria in our study population (≥24 months of remission on TNF-α inhibitors) and real-time recording of subclinical activity may have contributed to the apparent earlier median flare time. These factors underscore the importance of considering study methodology when comparing flare kinetics across cohorts.

### 3.2. Clinical Phenotype and Manifestations of Flare

Arthritis was the predominant manifestation of relapse, occurring in 50 of 58 flares (86%); uveitis affected seven patients (12%), and combined arthritis and uveitis occurred in 1 patient (2%). The pattern of joint involvement during flares largely recapitulated prior disease distribution: in 49/58 (84.4%) patients, the same joints were affected as before withdrawal. The knee (43/51; 84%) and ankle (25/51; 49%) were the most frequently involved joints, and the median number of active joints at flare (2; IQR 1–7) was significantly lower than at disease onset (median 7; IQR 2–7), indicating that post-withdrawal relapses tended to be milder in terms of joint count than the initial disease presentation. 58% of patients with a history of uveitis flared after withdrawal.

From a safety and management perspective, these results are reassuring in two respects. First, despite the high relapse frequency, most flares were limited in joint number and thus remained clinically manageable. Second, the reintroduction of therapy was effective: 56 of 58 patients who flared received pharmacologic retreatment, and 54 (96.4%) resumed the same TNF-α inhibitor. All retreated patients achieved clinical inactive disease per the C. Wallace criteria within six months. This high response rate to retreatment supports the reversibility of most withdrawal-related relapses when promptly managed.

### 3.3. Predictors of Sustained Remission and Flare—Integration with Broader Literature

The identification of predictors for disease relapse after withdrawal of biologic therapy in juvenile idiopathic arthritis (JIA) has been the subject of extensive investigation. According to multiple studies, patients with polyarticular disease, positive rheumatoid factor (RF) or antinuclear antibodies (ANA), concomitant uveitis, and elevated serum S100 protein concentrations are at higher risk of disease flare following biologic discontinuation [8,9,10]. In addition, imaging-detected subclinical synovitis on MRI or ultrasound (US) has also been associated with increased relapse risk [11,12,13,14].

Our study expands on these findings by systematically evaluating predictors of both sustained biologic-free remission and relapse in patients with non-systemic JIA who had achieved remission on TNF-α inhibitors. Factors associated with prolonged biologic-free remission, as defined by Wallace criteria and JADAS71 scores at 6, 12, and 18 months after TNF-α inhibitor withdrawal, included female sex, attainment of ≥90% improvement according to ACRpedi criteria at both 6 and 12 months, ANA negativity at biologic initiation, absence of *HLA-B27*, and continuation of methotrexate following biologic discontinuation. Conversely, predictors of disease flare without systemic manifestations included a CHAQ score of 2 or higher at disease onset (parental version), elevated serum S100 protein and high-sensitivity C-reactive protein (hsCRP) levels at the time of withdrawal, imaging evidence of subclinical synovitis by ultrasound or MRI, and a history of JIA-associated uveitis.

Several prior studies provide context for these observations. Lovell et al. (2018) identified longer disease duration at study entry, early-onset disease, a longer interval from diagnosis to inactive disease, and a shorter duration of inactive disease prior to TNF-α inhibitor cessation as risk factors for relapse [5].

Simonini et al. reported that sustained biologic-free remission was more likely in patients with systemic JIA and those receiving biologic therapy for more than two years. In contrast, patients with polyarticular or oligoarticular disease and less than two years of biologic treatment had a higher flare risk following withdrawal [15]. In contrast, our findings indicate that neither disease duration prior to treatment initiation nor JIA subtype had a statistically significant impact on the likelihood of maintaining biologic-free remission, suggesting that treatment response characteristics may be more informative than baseline disease phenotype in predicting post-withdrawal outcomes.

Consistent with Su et al. (2017), prolonged time from etanercept initiation to achievement of remission (14.9 ± 6.9 vs. 8 ± 1.2 months; *p* < 0.001) and longer duration of inactive disease (8.9 ± 6.9 vs. 2.0 ± 1.2 months; *p* < 0.001) were associated with higher flare risk [16].

Conversely, Cai et al. (2013) found no significant association between flare risk and sex, age at onset, disease duration, JIA subtype, DMARD co-therapy, *HLA-B27* status, presence of joint erosions, or duration of etanercept therapy [17].

Uveitis remains a particular concern. Simonini et al. (2018) demonstrated that patients with a history of JIA-associated chronic idiopathic uveitis were at elevated risk of ocular relapse after withdrawal of methotrexate or biologic therapy, with relapse particularly associated with slow attainment of inactive disease (>6 months) and ANA positivity [15].

Similarly, in our study population, 58% of patients with prior uveitis experienced relapse after TNF-α inhibitor cessation. Saboo et al. (2013) also reported that younger age at initiation of immunosuppressive therapy and longer intervals from disease onset to treatment initiation were associated with higher uveitis relapse risk [18].

Collectively, these findings underscore the need for individualized management strategies, including consideration of prolonged or indefinite biologic therapy and close ophthalmologic monitoring, in patients at high risk.

Beyond previously established predictors, our study identified novel indicators of relapse and prolonged remission. Specifically, early disease severity as measured by CHAQ ≥ 2 at onset, and a twofold increase in hsCRP at the time of biologic withdrawal, were independently associated with higher flare risk. Conversely, deep clinical response (ACRpedi ≥ 90 at 6 and 12 months) predicted prolonged biologic-free remission, highlighting the prognostic value of early treatment response. Notably, the interpretation of ANA negativity differed from prior literature. While previous reports emphasized historical ANA status, in our study population, ANA negativity at the time of biologic initiation was more predictive of sustained remission [9].

The prognostic significance of subclinical synovitis remains a topic of ongoing debate. Some studies have reported that imaging-detected synovial abnormalities in clinically inactive patients do not consistently predict flares (Magni-Manzoni et al., 2013 [19]), whereas other studies suggest a predictive role [12,19]. In our study population, more than half of patients with subclinical synovitis on US or MRI experienced a flare following TNF-α inhibitor withdrawal, supporting the potential utility of imaging in personalized tapering decisions [14,20].

Taken together, our findings provide a comprehensive, integrative perspective on predictors of flare and sustained biologic-free remission in non-systemic JIA. They corroborate previously reported risk factors, identify new, clinically actionable indicators, and emphasize the importance of early and deep clinical response, ongoing DMARD therapy, and individualized monitoring of high-risk subgroups, particularly those with a history of uveitis. These data may inform clinical decision-making, optimize timing of biologic tapering, and improve the likelihood of maintaining long-term remission without biologic therapy.

Beyond current biologic tapering strategies, our findings highlight the need for more precise and mechanism-based approaches to pharmacotherapy in non-systemic JIA. The observed heterogeneity of flare risk, including relapse in some patients without overt imaging inflammation and sustained remission in others with subclinical synovitis, suggests that systemic cytokine blockade alone may be insufficient to fully capture disease biology. Emerging research directions include targeting tissue–immune interaction pathways, modulating the local joint microenvironment, and developing therapies directed at specific inflammatory cell subtypes involved in synovial persistence. In this context, novel molecular approaches, such as nucleic acid–based therapeutics targeting defined inflammatory pathways or cell populations, have been proposed as a promising strategy for immune-mediated inflammatory diseases. Advances in targeted delivery systems and microenvironment-responsive therapies may enable more individualized treatment selection, improve long-term disease control, and reduce overtreatment. Such approaches warrant further investigation in JIA to complement existing biologic and DMARD-based strategies and to optimize precision medicine in pediatric rheumatology [21,22].

In 2024, Sabuh Sagheer et al. reported recent data in adult rheumatoid arthritis, suggesting that subcutaneous methotrexate administration may improve tolerability and that modulation of IL-2–dependent pathways could help control persistent inflammation. Although derived from adult RA, these findings provide mechanistic insights relevant to future individualized treatment strategies in juvenile idiopathic arthritis [23].

In this broader context, recent advances in inflammatory arthritis research further support the relevance of optimizing conventional immunomodulatory strategies alongside biologic therapies. For example, studies in rheumatoid arthritis have demonstrated that alternative methotrexate delivery routes, such as subcutaneous administration, may improve treatment efficacy and tolerability, while modulation of cytokine pathways, including IL-2–related mechanisms affecting regulatory T-cell balance, represents a promising adjunctive approach to controlling chronic joint inflammation. Although derived from adult RA models, these concepts underscore the importance of integrated, mechanism-informed treatment strategies that may also improve long-term disease control and treatment individualization in juvenile idiopathic arthritis.

### 3.4. Implications for Tapering Strategy and Personalization

Although we did not find a clear superiority of one tapering method over another, the identified predictors enable a risk-stratified, hypothesis-driven clinical approach. Patients with absent subclinical inflammation, low inflammatory biomarkers, a deep early response, and continued methotrexate may be considered better candidates for a withdrawal attempt. In contrast, those with CHAQ ≥ 2 at onset, elevated S100/hsCRP, positive *HLA-B27*, or a history of prior uveitis should be counseled regarding a high relapse risk and the need for close monitoring. Crucially, any algorithm derived from our data must be viewed as exploratory and require prospective validation. Interaction tests and stratified analyses with adequate power would be needed to justify the firm assignment of specific tapering modalities to particular subgroups.

### 3.5. Personalized Approach to TNF-α Inhibitor Discontinuation in Non-Systemic JIA

Based on the findings of this study, we developed a preliminary framework that may assist in considering TNF-α inhibitor withdrawal in patients with non-systemic JIA who have achieved sustained clinical remission. This algorithm is intended as a hypothesis-generating tool and should not be regarded as a definitive guideline; external validation in larger prospective cohorts is required.

#### 3.5.1. Step 1. Confirmation of Remission

Withdrawal should be considered only after inactive disease has been confirmed according to the C. Wallace criteria while the patient is still receiving TNF-α inhibitor therapy.

#### 3.5.2. Step 2. Pre-Withdrawal Evaluation

Before discontinuation, a structured clinical, laboratory, and imaging assessment may help estimate the probability of maintaining remission. The following factors were associated with remission outcomes in our study population and may be taken into account:

- History of JIA-associated uveitis;
- Achievement of ≥90% improvement by ACRpedi criteria at both 6 and 12 months after TNF-α inhibitor initiation;
- Baseline Childhood Health Assessment Questionnaire (CHAQ) score;
- Serum S100 protein concentration at withdrawal (threshold: <2.9 µg/L in our dataset);
- Serum high-sensitivity CRP (hsCRP) level at withdrawal (<5 mg/L);
- Presence or absence of subclinical synovitis on ultrasound or MRI.

#### 3.5.3. Step 3. Choice of Withdrawal Strategy

Three main approaches to withdrawal were evaluated in this study: abrupt discontinuation, gradual interval extension, and gradual dose reduction. While no single strategy demonstrated universal superiority, exploratory analyses suggested that patient-specific characteristics (e.g., sex, ANA and *HLA-B27* status, concomitant methotrexate therapy) might influence outcomes. At present, such associations should be viewed as preliminary and require further validation before firm recommendations can be made.

#### 3.5.4. Step 4. Monitoring During Withdrawal

For patients undergoing interval extension or dose reduction, complete cessation may be attempted if remission is sustained for at least three months under the modified regimen. Close monitoring during and after tapering is essential.

#### 3.5.5. Step 5. Management of Relapse

If a flare occurs following withdrawal, reintroduction of the previously effective TNF-α inhibitor is advisable. In our study population, most patients who relapsed regained inactive disease within six months of retreatment. If treatment targets are not met, therapeutic adjustments should be made in accordance with current international recommendations.

## 4. Methods

### 4.1. Study Design and Participants

This prospective, randomized, open-label, single-center study was conducted at the Pediatric Rheumatology Department of the National Medical Research Center for Children’s Health (Moscow, Russia). A total of 76 children with non-systemic juvenile idiopathic arthritis (JIA) in clinical remission for ≥24 months while receiving TNF-α inhibitor therapy (etanercept or adalimumab) were enrolled.

At TNF-α inhibitor initiation, 68 of 76 patients (89.5%) received concomitant disease-modifying antirheumatic drugs (DMARDs). Methotrexate was prescribed in most cases (64/68; 94.1%). By the time TNF-α inhibitors were withdrawn, 45 patients (59.2%) continued DMARDs (most commonly methotrexate). Discontinuation of DMARDs during follow-up was permitted only in the event of adverse events. The introduction of new immunosuppressive agents was not permitted.

### 4.2. Pre-Withdrawal Assessment and Follow-Up

Before treatment discontinuation, all patients underwent standardized screening for subclinical disease activity, including serum S100 proteins, high-sensitivity C-reactive protein (hsCRP). Serum S100A8/A9 and hsCRP levels were measured in a single laboratory using standardized commercial ELISA kits (Bühlmann MRP8/14 ELISA; Biomerica, Inc., Irvine, CA, USA). Musculoskeletal ultrasound (US), and magnetic resonance imaging (MRI) of previously affected joints. All MRI examinations were performed on GE Healthcare scanners with field strengths of 1.5 T and 3 T and included standard anatomical and fluid-sensitive sequences, with contrast-enhanced imaging performed in all cases to assess synovial inflammation. After withdrawal, clinical and laboratory assessments were performed at 3, 6, 9, 12, and 18 months. Data were collected at disease onset, at the initiation of TNF-α inhibitors, every 6 months during therapy, at withdrawal, and during follow-up visits.

### 4.3. Randomization and Withdrawal Strategies

Patients were randomized in a 1:1:1 ratio using a computer-generated randomization sequence created by a study investigator. Allocation concealment was ensured by the use of sequentially numbered, opaque, sealed envelopes prepared in advance and opened only after patient enrollment and written informed consent. Neither the treating physicians nor the patients were aware of the upcoming group assignment prior to envelope opening. No crossover between withdrawal strategies was allowed after randomization, and all patients remained in their originally assigned tapering arms. Patients were allocated into three groups with predefined withdrawal strategies: Group I (abrupt withdrawal): immediate discontinuation of the TNF-α inhibitor; Group II (interval extension): gradual extension of dosing intervals (etanercept 0.8 mg/kg every two weeks instead of weekly; adalimumab 24 mg/m^2^ every four weeks instead of every two weeks), with intervals doubled until withdrawal if remission persisted; and Group III (dose reduction): gradual tapering of the dose while maintaining injection frequency (etanercept 0.4 mg/kg weekly; adalimumab 12 mg/m^2^ every two weeks). In Groups II and III, biologic therapy was discontinued entirely if remission persisted for at least 3 months after dose modification (Table 3).

### 4.4. Assessments, Outcomes, and Definitions

Variables included demographic characteristics (sex, age), disease-related features (JIA category, disease duration, remission duration, active joint count, history of uveitis, presence of systemic features), treatment response (degree of clinical improvement according to the American College of Rheumatology Pediatric (ACRpedi)] response criteria at 6 and 12 months after TNF-α inhibitor initiation, laboratory biomarkers [erythrocyte sedimentation rate (ESR), C-reactive protein (CRP), antinuclear antibodies (ANA), human leukocyte antigen B27 (*HLA-B27*), S100 proteins (serum calprotectin), high-sensitivity C-reactive protein (hsCRP)], imaging markers [subclinical synovitis on musculoskeletal ultrasound (US) or magnetic resonance imaging (MRI)], and functional outcomes [visual analog scale (VAS), Childhood Health Assessment Questionnaire (CHAQ), ACRpedi].

Clinical remission and flare were defined according to the C. Wallace criteria [24]. Non-biologic remission was defined as ≥12 months of sustained remission after withdrawal of TNF-α inhibitors, with or without DMARDs (excluding corticosteroids and biologics). For clarity, in the present study, the term “sustained biologic-free remission” refers exclusively to remission maintained for at least 12 months after TNF-α inhibitor withdrawal, as defined by the Wallace criteria. Assessments at 6 and 18 months are reported as follow-up time points. Transient synovitis resolving with nonsteroidal anti-inflammatory drugs (NSAIDs) before the next visit was not considered a flare.

Primary endpoint: sustained biologic-free remission at 12 months after TNF-α inhibitor withdrawal, defined according to the C. Wallace criteria and JADAS71.

Secondary endpoints: biologic-free remission status at 6 months (interim follow-up assessment) and at 18 months (long-term follow-up); time to disease flare; clinical features of relapse (joint count, pattern, uveitis); biochemical and imaging markers at withdrawal (S100 protein, hsCRP, subclinical synovitis on US/MRI); response to retreatment; and identification of predictors of sustained remission and flare.

### 4.5. Predictors of Remission

**Figure 3:**
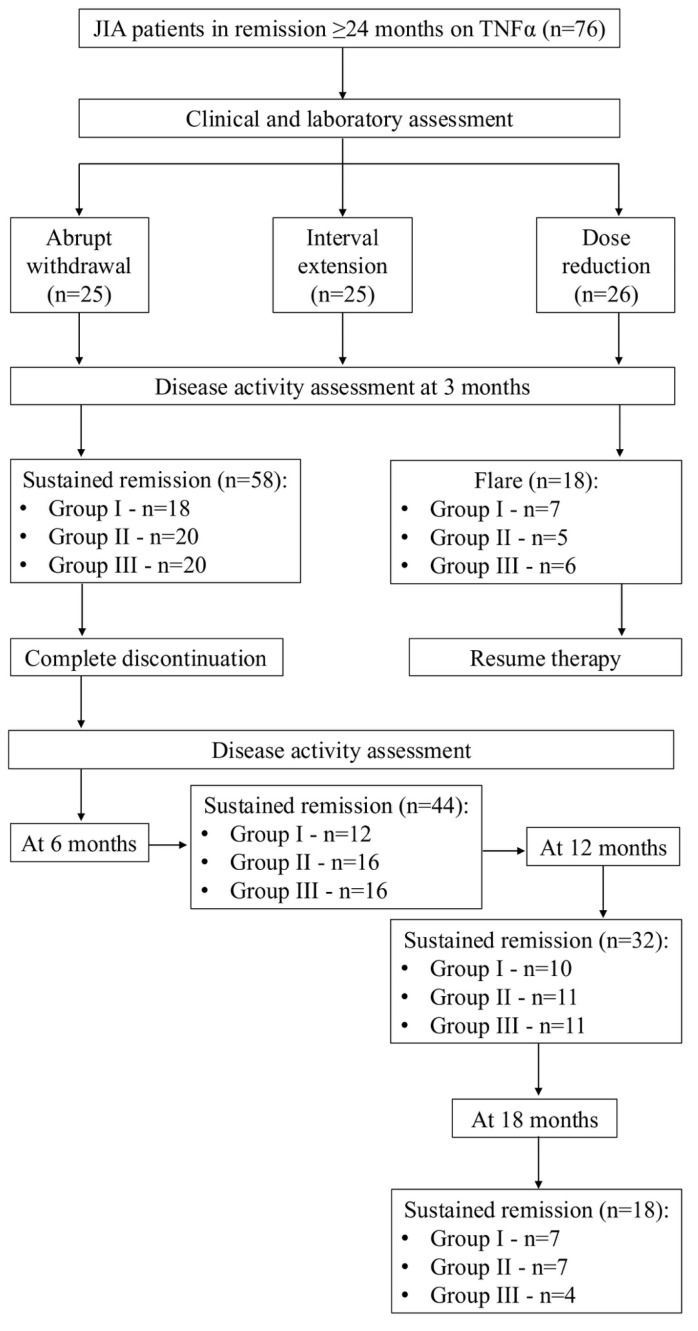
Flow diagram of the TNF-α inhibitor withdrawal study.

To identify predictors of sustained biologic-free remission, 99 clinical, laboratory, and imaging parameters were analyzed using binary logistic regression. Variables with *p* < 0.10 in univariate analysis were entered into multivariate models; *p* < 0.05 was considered statistically significant (Figure 3).

### 4.6. Statistical Analysis

Statistical analysis and data visualization were performed using the R statistical computing environment, version 4.2.2 (R Foundation for Statistical Computing, Vienna, Austria).

Prior to the prospective phase of the study, a sample size calculation was performed. The study planned to enroll 174 patients, with 58 per group. However, it was not possible to reach this target due to strict inclusion criteria.

Descriptive statistics are presented as counts and relative frequencies for categorical variables, mean (standard deviation) and median (first and third quartiles) for symmetrically distributed continuous variables, and median (first and third quartiles) for asymmetrically distributed continuous variables. The distributions’ skewness was assessed using the skewness coefficient; values greater than 1.96 were considered indicative of significant asymmetry.

Group comparisons for categorical variables were performed using Fisher’s exact test. For continuous variables, one-way analysis of variance was used for symmetrically distributed data, and the Kruskal–Wallis test was used for asymmetrically distributed data, with Dunn’s test as a post hoc test. Holm’s procedure was applied to adjust *p*-values for multiple testing. Statistical significance was defined as *p* < 0.05.

Binary logistic regression models were employed to assess the relationships between potential predictors and study outcomes. Odds ratios (ORs) with corresponding 95% confidence intervals (95% CIs) were reported as measures of association. Separate logistic regression models were constructed to identify predictors of sustained biologic-free remission and predictors of disease flare. Quantitative covariates with pronounced skewness were log2-transformed before being included in the models. To assess differences in direction and strength of associations across groups, interaction terms were included in logistic regression models. Associations were considered statistically significant at *p* < 0.05.

For time-to-event analyses, Kaplan–Meier estimates, the log-rank test, and univariate Cox proportional hazards models were used, with hazard ratios (HRs) and Harrell’s C-index (analogous to AUC) reported. Associations were considered significant at *p* < 0.05.

Stepwise selection based on the Akaike information criterion (AIC) was applied to build multivariate models, including predictors with *p* < 0.2 in univariate analyses. Model performance was evaluated using Harrell’s C-index and the Nagelkerke pseudo-R^2^.

## 5. Limitations

Several limitations of this study should be acknowledged. First, clinical assessments and flare evaluations were not blinded to the withdrawal strategy. Although evaluations were performed by a consensus of the treating physician, the head of the department, and the study investigator, rather than by a single assessor, the lack of blinded or duplicate review may have introduced observer bias. In addition, part of the data was extracted from electronic medical records, which may be subject to incomplete documentation or variability in routine clinical practice.

Second, the strict inclusion criterion requiring at least 24 months of sustained remission on TNF-α inhibitor therapy substantially limited patient recruitment, resulting in a final sample size of 76 patients, which fell short of the initially calculated target of 174. This reduces statistical power, particularly in multivariable analyses, and increases the risk of overfitting and instability in effect estimates. Consequently, analyses identifying predictors of sustained biologic-free remission and disease flare should be interpreted as exploratory, and residual confounding by unmeasured factors cannot be excluded. The proposed withdrawal algorithm should therefore be considered hypothesis-generating rather than definitive.

Third, imaging assessments were performed at a single time point prior to biologic withdrawal and were limited to previously affected joints rather than a standardized joint set. The radiologist’s opinion was not re-evaluated, and the images were not double-blinded assessed. This approach may underestimate the extent of subclinical inflammation and does not capture its dynamic evolution over time. Although serum S100 (MRP8/14) and hsCRP were measured using standardized commercial assays in a single laboratory, these biomarkers reflect single measurements and may not fully represent longitudinal inflammatory activity.

In gradual tapering strategies, the initiation of withdrawal does not coincide with complete biologic cessation, which should be considered when interpreting time-to-flare analyses.

Finally, the single-center design and inclusion of patients from a tertiary referral center in Russia may limit the generalizability of the findings to other ethnic, socio-economic, and healthcare settings.

Despite these limitations, this prospective study provides clinically relevant data on flare rates, timing, and predictors following TNF-α inhibitor withdrawal in non-systemic JIA. The results highlight that even patients with prolonged remission remain at substantial risk of relapse after biologic cessation. Integration of clinical characteristics, laboratory biomarkers, and imaging findings may support individualized tapering strategies and optimized post-withdrawal monitoring to mitigate flare risk.

## 6. Conclusions

In this prospective study, the population of children with non-systemic JIA, withdrawal of TNF-α inhibitors after ≥24 months of sustained remission was associated with a high risk of disease flare, with most relapses occurring within the first year. Flare manifestations were predominantly articular, generally milder than those at initial disease onset, and largely reversible with the prompt reintroduction of therapy.

Multivariable analysis identified key predictors of biologic-free remission and flare. Sustained remission was associated with female sex, deep early response (ACRpedi ≥ 90% at 6 and 12 months), ANA negativity at biologic initiation, absence of *HLA-B27*, and continued methotrexate therapy after withdrawal. Conversely, higher CHAQ scores at disease onset, elevated serum S100 protein and hsCRP at withdrawal, imaging evidence of subclinical synovitis, and a history of JIA-associated uveitis were linked to increased flare risk. These findings underscore the value of integrating clinical, laboratory, and imaging parameters to inform personalized tapering decisions.

However, given the limited sample size and the exploratory nature of the predictive analyses, these findings and the proposed withdrawal algorithm should be interpreted with caution.

Despite the identification of predictors for sustained remission, cessation of TNF-α inhibitors remains a high-risk intervention. Careful patient selection, individualized risk assessment, and close monitoring are crucial for optimizing outcomes and minimizing flare-related morbidity. Future studies with larger cohorts and standardized imaging and biomarker protocols are warranted to validate these predictors and refine strategies for safe biologic tapering in JIA. Our findings emphasize that biologic withdrawal should be guided primarily by individual disease biology rather than by the tapering strategy itself.

## Figures and Tables

**Table 1:** Characteristics of the 76 JIA patients who were in remission or flared after anti-TNFα therapy withdrawal.

Characteristics	Total, *n* = 76	Withdrawal Strategies	Withdrawal Strategies	Withdrawal Strategies	Withdrawal Strategies
		Abrupt Discontinuation *n* = 25	Gradual Interval Extension *n* = 25	Gradual Dose Reduction *n* = 26	*p*
Gender, *n* (%) Male Female	29 (38.2) 47 (61.8)	18 (72) 7 (28)	14 (56) 11 (44)	15 (57.7) 11 (42.3)	<0.05
Age at disease onset, years Me (25%; 75%)	3.6 (1.8; 5.8)	3.9 (2.0; 5.7)	3.6 (1.7; 5.5)	3.0 (1.7; 6.1)	ns
Age at start of anti-TNFα, years, Me (25%; 75%)	6.6 (4.1; 8.6)	6.6 (3.0; 8.2)	6.2 (4.2; 7.8)	7.1 (3.8; 10.3)	ns
JIA category, *n* (%) Persistent oligoarticular RF-negative polyarticular Enthesitis-related Extended oligoarticular	26 (34.2) 17 (22.3) 17 (22.3) 16 (21)	7 (28) 8 (32) 4 (16) 6 (24)	7 (28) 6 (24) 6 (24) 6 (24)	11 (42) 4 (15) 7 (26) 4 (15)	ns
Duration of disease prior to TNF-α inhibitors, months, Me (25%; 75%)	19 (9; 45)	19 (8.1; 19.2)	22 (10; 29)	21 (11; 28)	ns
Active joints *, Me (25%; 75%)	7 (2; 7)	5 (2; 8)	4 (4; 2)	4 (2; 6)	ns
Swollen joints *, Me (25%; 75%)	4 (2; 4)	3 (2; 6)	3 (2; 4)	3 (2; 4)	ns
Painful joints *, Me (25%; 75%)	3 (2; 4)	4 (2; 6)	3 (2; 4)	3 (2; 4)	ns
Joints with limited motion *, Me (25%; 75%)	4 (2; 4)	4 (2; 6)	2 (2; 4)	4 (2; 4)	ns
Morning stiffness, min *, Me (25%; 75%)	38 (0; 60)	30 (0; 130)	0 (0; 60)	0 (0; 60)	ns
Active uveitis *, *n* (%)	8 (10.5)	2 (8)	4 (16)	2 (7.8)	<0.01
ESR *, mm/hour, Me (25%; 75%)	18 (6; 28)	18 (6; 28)	18 (6; 26)	20 (7; 29)	ns
CRP *, mg/L, Me (25%; 75%)	6 (2; 15)	6 (3; 12)	6 (2; 12)	6 (2; 17)	ns
hsCRP, mg/L, Me (25%; 75%)	1.1 (0.1; 7.13)	1.3 (0.1; 5.9)	1.3 (0.1; 7.13)	0.5 (0.1; 3.09)	<0.01
S100 protein, µg/L, Me (25%; 75%)	1.5 (0.4; 6.7)	1.4 (0.4; 5)	1.5 (0.4; 4.3)	2.1 (0.4; 6.69)	<0.01
ANA positivity, *n* (%)	42 (55.3)	16 (64)	12 (48)	14 (53)	<0.05
HLA-B27 positive, *n* (%)	18 (23.7)	5 (20)	5 (20)	8 (30)	<0.05
MDVAS *, mm, Me (25%; 75%)	85 (83; 90)	80 (80; 82.5)	84 (84; 88)	84 (83; 89)	ns
MRI findings, *n* (%) Subclinical synovitis Tenosynovitis Bone marrow edema Pannus	45 (59.2) 5 (6.6) 11 (14.5) 6 (7.9)	15 (60) 3 (12) 1 (4) 2 (8)	13 (52) 2 (8) 5 (20) 1 (4)	17 (65.4) 0 5 (19.2) 3 (11.5)	<0.001
Ultrasound findings, *n* (%) Subclinical synovitis Pannus	45 (59.2) 5 (6.6)	15 (60) 1(4)	13 (52) 1 (4)	17 (65.4) 3 (1.5)	<0.001
Concomitant treatment *, *n* (%) methotrexate sulphasalazine methotrexate + cyclosporine A tofacitinib	68 (89.5) 64 (94.1) 1 (1.5) 3 (4.4) 1(1.5)	24 (96) 22 (91) 1 (4) 1 (4) 0	22 (88) 20 (90) 0 1 (4) 1 (4)	23 (88) 22 (95) 0 1 (4.5) 0	ns
Biologic “naive” patients, no. (%)	71 (93.4)	25 (100)	20 (80)	26 (100)	ns
Previous biologic therapy, no. (%) infliximab tocilizumab abatacept certolizumab	5 (7.9) 2 (2.6) 1 (1.3) 1 (1.3) 1 (1.3)	0	5 (20) 2 (8) 1 (4) 1 (4) 1 (4)	0	ns
Anti-TNF-α treatment, *n* (%) etanercept adalimumab	67 (88) 9 (11.8)	22 (88) 3 (12)	20 (80) 5 (20)	25 (96) 1 (4)	ns
Concomitant treatment **, no. (%) methotrexate leflunomide cyclosporine A	45 (59.2) 43 (95.6) 1 (2.2) 1 (2.2)	14 (56) 13 (92.9) 1 (7.1) 0	15 (60) 14 (93.3) 0 1 (6.7)	16 (61.5) 16 (100) 0 0	<0.05

* At the time of TNF-α inhibitor start. ** At the time of TNF-α inhibitor discontinuation. ANA—antinuclear antibody test, TNF—tumor necrosis factor, RF—rheumatoid factor, ESR—erythrocyte sedimentation rate, CRP—C-reactive protein, JIA—juvenile idiopathic arthritis, HLA-B27—human leukocyte antigen B27; ns—not significant.

**Table 2:** Predictors of sustained biologic-free remission after TNF-α inhibitor withdrawal.

Predictors of Sustained Drug-Free Remission	Predictors of Sustained Drug-Free Remission	Predictors of Sustained Drug-Free Remission	Predictors of Sustained Drug-Free Remission
Female sex	Increased likelihood of sustained remission	7.0 (1.3–47.6)	<0.05
ACRpedi ≥ 90% improvement at 6 and 12 months	Increased likelihood of sustained remission	2.33 (1.88–6.30)	<0.001
ANA negativity at TNF-α inhibitor initiation	Increased likelihood of sustained remission	1.7 (1.19–2.39)	<0.05
Absence of HLA-B27	Increased likelihood of sustained remission	3.4 (1.3–8.6)	<0.01
Ongoing methotrexate therapy after withdrawal	Increased likelihood of sustained remission	3.17 (1.23–8.53)	<0.05
Predictors of disease flare	Predictors of disease flare	Predictors of disease flare	Predictors of disease flare
CHAQ score ≥ two at disease onset	Increased risk of flare	1.56 (1.3–2.0)	<0.01
Elevated serum S100 protein at withdrawal	Increased risk of flare	1.81 (1.07–3.7)	<0.05
Elevated hsCRP at withdrawal	Increased risk of flare	1.34 (1.1–1.7)	<0.05
Subclinical synovitis on US or MRI	Increased risk of flare	6.67 (3.4–13.0)	<0.0001
History of JIA-associated uveitis	Increased risk of flare	2.77 (1.72–4.48)	<0.05

**Table 3:** Strategies for TNF-α inhibitor discontinuation.

I	Abrupt Withdrawal	Immediate Cessation (0.8 mg/kg/week)	Immediate Cessation (24 mg/m^2^ Every 2 Weeks)	Not Applicable
II	Interval extension	0.8 mg/kg every 2 weeks → every 4 weeks → stop	24 mg/m^2^ every 4 weeks → every 8 weeks → stop	Sustained remission for 3 months after interval change
III	Dose reduction	0.4 mg/kg weekly → stop	12 mg/m^2^ every 2 weeks → stop	Sustained remission for 3 months after dose reduction

## Data Availability

The datasets generated during and/or analyzed during the current study are available from the corresponding author upon reasonable request and subject to institutional approval. The data are not publicly available due to ethical and privacy restrictions related to patient confidentiality.

## References

[1] Long A.M., Marston B. (2023). Juvenile Idiopathic Arthritis. Pediatr. Rev..

[2] Beresford M.W., Baildam E.M. (2009). New advances in the management of juvenile idiopathic arthritis—2: The era of biologicals. Arch. Dis. Child. Educ. Pract. Ed..

[3] Pedersen M.L., Neve-Græsbøll A., Herlin T., Glerup M. (2023). Biologic switching patterns among children with non-systemic juvenile idiopathic arthritis. Pediatr. Rheumatol..

[4] Gieling J., van den Bemt B., Hoppenreijs E., Schatorjé E. (2022). Discontinuation of biologic DMARDs in non-systemic JIA patients: A scoping review of relapse rates and associated factors. Pediatr. Rheumatol. Online J..

[5] Lovell D.J., Johnson A.L., Huang B., Gottlieb B.S., Morris P.W., Kimura Y., Onel K., Li S.C., Grom A.A., Taylor J. (2018). Risk, timing, and predictors of disease flare after discontinuation of anti-tumor necrosis factor therapy in children with polyarticular forms of juvenile idiopathic arthritis with clinically inactive disease. Arthritis Rheumatol..

[6] Halyabar O., Mehta J., Ringold S., Rumsey D.G., Horton D.B. (2019). Treatment withdrawal following remission in juvenile idiopathic arthritis: A systematic review of the literature. Paediatr. Drugs.

[7] Alexeeva E.I., Tsulukiya I.T., Dvoryakovskaya T.M., Kudlay D.A., Kriulin I.A., Botova M.S., Kondratyeva N.M., Krekhova E.A., Shingarova M.S., Kokina M.Y. (2025). Can we successfully discontinue anti-tumor necrosis factor-α treatment in children with non-systemic juvenile idiopathic arthritis? The experience of a tertiary center. Biomedicines.

[8] Anink J., Van Suijlekom-Smit L.W.A., Otten M.H., Prince F.H.M., van Rossum M.A.J., Dolman K.M., Hoppenreijs E.P.A.H., Cate R.T., Ursu S., Wedderburn L.R. (2015). MRP8/14 serum levels as a predictor of response to starting and stopping anti-TNF treatment in juvenile idiopathic arthritis. Arthritis Res Ther..

[9] Aquilani A., Marafon D.P., Marasco E., Nicolai R., Messia V., Perfetti F., Magni-Manzoni S., De Benedetti F. (2018). Predictors of flare following etanercept withdrawal in patients with rheumatoid factor-negative juvenile idiopathic arthritis who reached remission while taking medication. J. Rheumatol..

[10] Simonini G., Bracaglia C., Cattalini M., Taddio A., Brambilla A., De Libero C., Marafon D.P., Caputo R., Cimaz R. (2017). Predictors of relapse after discontinuing systemic treatment in childhood autoimmune chronic uveitis. J. Rheumatol..

[11] van Gulik E.C., Hemke R., Welsink-Karssies M.M., Schonenberg-Meinema D., Dolman K.M., Barendregt A.M., Nusman C.M., Maas M., Kuijpers T.W., Berg J.M.v.D. (2018). Normal MRI findings of the knee in patients with clinically active juvenile idiopathic arthritis. Eur. J. Radiol..

[12] De Lucia O., Ravagnani V., Pregnolato F., Hila A., Pontikaki I., Gattinara M., Romano M., Gerloni V., Pieropan S., Murgo A. (2018). Baseline ultrasound examination as possible predictor of relapse in patients affected by juvenile idiopathic arthritis (JIA). Ann. Rheum. Dis..

[13] Munir S., Patil K., Miller E., Uleryk E., Twilt M., Spiegel L., Doria A.S. (2014). Juvenile idiopathic arthritis of the axial joints: A systematic review of the diagnostic accuracy and predictive value of conventional MRI. AJR Am. J. Roentgenol..

[14] Zhao Y., Rascoff N.E., Iyer R.S., Thapa M., Reichley L., Oron A.P., Wallace C.A. (2018). Flares of disease in children with clinically inactive juvenile idiopathic arthritis were not correlated with ultrasound findings. J. Rheumatol..

[15] Simonini G., Ferrara G., Pontikaki I., Scoccimarro E., Giani T., Taddio A., Meroni P.L., Cimaz R. (2018). Flares after withdrawal of biologic therapies in juvenile idiopathic arthritis: Clinical and laboratory correlates of remission duration. Arthritis Care Res..

[16] Su Y., Yang Y.H., Chiang B.L. (2017). Treatment response to etanercept in methotrexate-refractory juvenile idiopathic arthritis: An analysis of predictors and long-term outcomes. Clin. Rheumatol..

[17] Cai Y., Liu X., Zhang W., Xu J., Cao L. (2013). Clinical trial of etanercept tapering in juvenile idiopathic arthritis during remission. Rheumatol. Int..

[18] Saboo U.S., Metzinger J.L., Radwan A., Arcinue C., Parikh R., Mohamed A., Foster C.S. (2013). Risk factors associated with the relapse of uveitis in patients with juvenile idiopathic arthritis: A preliminary report. J. AAPOS.

[19] Magni-Manzoni S., Scirè C.A., Ravelli A., Klersy C., Rossi S., Muratore V., Visconti C., Lanni S., Merli P., Montecucco C. (2013). Ultrasound-detected synovial abnormalities are frequent in clinically inactive juvenile idiopathic arthritis, but do not predict a flare of synovitis. Ann. Rheum. Dis..

[20] Miotto E Silva V.B., Mitraud S.A.V., Furtado R.N.V., Natour J., Len C.A., Maria Teresa de Sande E Lemos Ramos Ascensão Terreri (2017). Patients with juvenile idiopathic arthritis in clinical remission with positive power Doppler signal in joint ultrasonography have an increased rate of clinical flare: A prospective study. Pediatr. Rheumatol. Online J..

[21] Xu L., Shao Z., Fang X., Xin Z., Zhao S., Zhang H., Zhang Y., Zheng W., Yu X., Zhang Z. (2025). Exploring precision treatments in immune-mediated inflammatory diseases: Harnessing the infinite potential of nucleic acid delivery. Exploration.

[22] Li K., Zhu Y., Alini M., Stoddart M.J., Grad S., Li Z. (2024). Establishment of a coculture system with osteochondral and synovial explants as an ex vivo inflammatory osteoarthritis model. Eur. Cells Mater..

[23] Sagheer S., Rasheed M.S., Ashraf F., Rao A., Fatima M., Ajmal M.N., Ameen A., Mohamed K., Khan A., Ali F. (2024). Enhancing rheumatoid arthritis treatment by subcutaneous methotrexate injections and anti-IL-2 antibody synthesis. Int. J. Agric. Biosci..

[24] Wallace C.A., Ruperto N., Giannini E. (2004). Childhood Arthritis and Rheumatology Research Alliance; Pediatric Rheumatology International Trials Organization; Pediatric Rheumatology Collaborative Study Group. Preliminary criteria for clinical remission for select categories of juvenile idiopathic arthritis. J. Rheumatol..

